# Immunosuppressive Mechanisms in Brucellosis in Light of Chronic Bacterial Diseases

**DOI:** 10.3390/microorganisms10071260

**Published:** 2022-06-21

**Authors:** Joaquin Miguel Pellegrini, Jean-Pierre Gorvel, Sylvie Mémet

**Affiliations:** Centre d’Immunologie de Marseille Luminy, INSERM, CNRS, Aix-Marseille Université, Parc Scientifique et Technologique de Luminy, Case 906, CEDEX 09, 13288 Marseille, France; pellegrini@ciml.univ-mrs.fr (J.M.P.); gorvel@ciml.univ-mrs.fr (J.-P.G.)

**Keywords:** *Brucella*, chronic infection, persistence, immunosuppression, intracellular bacteria

## Abstract

Brucellosis is considered one of the major zoonoses worldwide, constituting a critical livestock and human health concern with a huge socio-economic burden. *Brucella* genus, its etiologic agent, is composed of intracellular bacteria that have evolved a prodigious ability to elude and shape host immunity to establish chronic infection. *Brucella*’s intracellular lifestyle and pathogen-associated molecular patterns, such as its specific lipopolysaccharide (LPS), are key factors for hiding and hampering recognition by the immune system. Here, we will review the current knowledge of evading and immunosuppressive mechanisms elicited by *Brucella* species to persist stealthily in their hosts, such as those triggered by their LPS and cyclic β-1,2-d-glucan or involved in neutrophil and monocyte avoidance, antigen presentation impairment, the modulation of T cell responses and immunometabolism. Attractive strategies exploited by other successful chronic pathogenic bacteria, including *Mycobacteria*, *Salmonella*, and *Chlamydia*, will be also discussed, with a special emphasis on the mechanisms operating in brucellosis, such as granuloma formation, pyroptosis, and manipulation of type I and III IFNs, B cells, innate lymphoid cells, and host lipids. A better understanding of these stratagems is essential to fighting bacterial chronic infections and designing innovative treatments and vaccines.

## 1. Introduction

135 years after the discovery of the etiological agent of the Malta fever by David Bruce and his team, brucellosis is still a worldwide and significant health problem, and the mechanisms that determine the establishment of chronic infection remain poorly understood. Brucellosis is a global re-emerging zoonosis that affects both livestock and wildlife [1]. Its socio-economic burden is huge, including losses due to brucellosis in livestock populations, amounting to billions of dollars per year, and healthcare and non-healthcare costs associated with human brucellosis [2]. Due to its high infectivity by some species, *Brucella*, its causative agent, has been classified as a potential warfare agent [3] and is manipulated in BSL3.

*Brucella* genus is composed of Gram-negative aerobic bacteria classified by microbiology methods and molecular taxonomy into different species according to their pathogenicity and host preference [4]. For example, *B. melitensis* infects preferentially goats and sheep, *B. abortus* cattle, *B. suis* swine, *B. ovis* sheep, *B. canis* dogs, *B. microti* common voles, and *B. neotomae* woodrats [1] and a diverse array of land and aquatic mammals as well as amphibians, as recently described [5]. *Brucella* species have a variable infectious dose in humans, depending on the strain, from very high to low [6].

Animal brucellosis in livestock animals is highly contagious and transmission is mainly driven by direct contact with other infected animals or their secretions (ingestion of contaminated placenta, aborted foetuses, contaminated milk, or breeding using contaminated semen) [7,8]. The main consequences of brucellosis in animals are abortion, metritis, reduced fertility, decreased milk production in females, and orchiepididymitis and infertility in males [4]. Occasionally, infected animals also develop hygromas (inflamed synovial bursae), which are potential sources of infection and cause articular pain.

*B. melitensis*, *B. abortus*, and *B. suis* are the main spp. responsible for human disease [2,9,10], transmitted via contaminated food or infected aerosol particles to people in close contact with infected animals. The acute phase is characterised by an undulant febrile illness, together with other non-specific symptoms that resemble those of a flu-like infection (sweats, malaise, arthralgia, lower back pain, and headache). These diverse manifestations complicate an accurate diagnosis and explain the generalised misdiagnosis and mistreatment [10,11], leading to an underestimation of the real impact of human brucellosis. When left untreated, as often the case, brucellosis leads to chronic inflammation [12], inducing a devastating multi-organ disease in humans with serious health complications. Chronic human brucellosis’ common sequelae comprise recurring febrile episodes combined with joint pain [13,14,15], and may evolve to severe forms associated with endocarditis, orchitis, spondylitis, osteomyelitis, arthritis, and meningoencephalitis [16].

Yet to date, there are no available vaccines against human brucellosis and only a few and ineffective approved vaccines to control brucellosis in livestock, which induce abortions in pregnant animals and are virulent for humans [17,18]. Moreover, anti-brucellosis treatment for humans consists of long-term therapies of combined antibiotics with significant failures and relapses.

Like *Brucella*, many bacteria persist in their hosts for protracted periods of time (i.e., *Salmonella*, *Mycobacteria*, *Chlamydia*, *Coxiella*), resulting in high levels of morbidity and mortality, and important economic losses all around the globe. Besides, long schemes or repetitive use of antibiotics have been suggested to lead to serious disturbances of intestinal microbiota [19] and to the emergence of antibiotic-resistant pathogenic strains. Considering their notable negative consequences, chronic infections have become a growing area of research, still with substantial gaps in our knowledge of their complete biology.

The progression into chronicity in both animals and humans is dictated by the ability of the bacteria to persist unnoticed for prolonged periods of time within host cells, and to resist and manipulate the host immune response in order to evade host elimination mechanisms [20,21]. Therefore, there is an urgent need for an in-depth dissection of the mechanisms involved in host immunity avoidance during bacterial persistence to design better vaccines and treatments in light of these major public health concerns. In this report, we summarize the current knowledge about the immunosuppressive mechanisms developed by *Brucella* to establish chronicity in its hosts and novel findings operating in other chronic bacterial diseases that would merit further investigation in the field.

## 2. Limiting Host Immunity to Establish Chronic *Brucella* Infection

*Brucella* ingresses into the host through the oropharyngeal and genital mucosal membranes and is rapidly internalised by professional phagocytes, such as macrophages, neutrophils, and dendritic cells (DC). These cells migrate to draining secondary and tertiary lymphoid organs [1,22], from where the bacteria disseminate to almost any organ into the body. In animals, *Brucella* has a strong tropism for reproductive organs, infecting preferentially placental trophoblasts [23,24,25,26] and mammary glands [27]. Different bacterial strategies have been described encompassing the breadth of *Brucella* pathobiology that allow successful persistence and are related to *Brucella’s* intracellular lifestyle, resistance, and subversion of host immune responses.

### 2.1. The Intracellular Lifestyle of Brucella

Although it may not be considered as an immunosuppressive mechanism per se, the intracellular journey of *Brucella* is critical to understanding later events affecting innate immunity, metabolism, antigen presentation, and adaptive response development, among others.

For thousands of years, *Brucella* has co-evolved with its mammalian hosts to exploit intracellular compartments of the endocytic, secretory, and autophagy pathways by using an array of Type IV secretion system (T4SS)-delivered effectors and other virulence factors. After phagocytosis, *Brucella* first resides within a membrane-bound vacuole, named *Brucella*-containing vacuole (BCV), which progressively interacts with early endosomes (as denoted by the presence of Rab5, EEA1, and the transferrin receptor, TfR) and late endosomes [26,28,29,30,31,32,33,34], acquiring markers, such as LAMP1, CD63, and Rab7, and reducing its pH to ~4–4.5, indicative of a normal maturation process. The BCVs can also interact with lysosomes [34].

Importantly, BCV acidification promotes the expression of the VirB Type IV secretion system and, therefore, the translocation of effector proteins (RicA and SepA, among others) that mediate BCV interactions with the endoplasmic reticulum (ER) exit site and the acquisition of ER and Golgi-derived membranes. This leads to the formation of a permissive ER-derived vacuole, named replicative BCVs (rBCVs), where bacteria extensively replicate. The induction of the unfolded protein response (UPR) has been suggested to promote rBCV biogenesis and bacterial replication [35,36,37]. At late stages of infection, *Brucella* translocates into vacuoles with autophagic features (aBCV), an essential step to complete its intracellular life cycle and facilitate reinfection events. This infers a bacterial manipulation of the autophagy machinery for subversion of host clearance and promotion of cell-to-cell spreading [38]. Likewise, the *Brucella* effector BspL was recently shown to hijack the ER-associated degradation (ERAD) components and delay the formation of the aBCV, thus allowing the bacteria extra time for extensive intracellular multiplication [39].

By controlling several cellular processes, *Brucella* co-opts its intracellular environment to ensure its persistence. Progression towards an ER-derived compartment provides protection from classical elimination mechanisms and gives the bacterium the conditions to replicate for long periods of time, ensuring nutrient acquisition and manipulation of cell death and metabolism. These events are essential for *Brucella*–host interactions and determine the type of immune response developed after the encounter with the bacteria; for instance, higher DC activation correlates with an inefficient ER replicative niche targeting of *Brucella* [40,41]. Thus, an in-depth knowledge of *Brucella*’ intracellular niche interactions and how *Brucella* antigens are processed and presented to T cells is, in this context, essential to designing successful vaccines and treatments.

### 2.2. Brucella Strategies for Hiding and Hampering Recognition

Historically, *Brucella* has been recognised as a stealthy pathogen, capable of hindering innate immune recognition, ultimately neglecting the generation of a proper adaptive immune response. *Brucella* is indeed devoid of classical pathogen-associated molecular patterns (PAMPs), such as capsules, fimbriae, and pili, and presents other PAMPs like Lipopolysaccharide (LPS) or outer membrane proteins with atypical features. The role of the Toll-like receptors (TLRs) in the resistance to *Brucella* infection has been extensively studied. *Brucella* recognition like that of any Gram-negative bacteria occurs via TLR2, TLR4, and TLR5, but is greatly reduced [42,43]. By using a systemic model of TLR knock-out mice infection via the intraperitoneal route, TLR2 and TLR4 were shown to be dispensable for an efficient elimination of *B. abortus* [44]. In contrast, the adaptor molecule MyD88 (involved in the signalling pathway of most TLRs as well as of interleukin (IL)-1β and IL-18), presented a delayed activation and was essential for inflammation and clearance of *Brucella* in vivo [44]. A similar observation has been made following aerosol challenge in mice [45], although these molecules seem to contribute to *Brucella* clearance from the lungs from week 4 onwards and also impact on antibody responses, suggesting a time and tissue-segregated relevance for TLR engagement. TLR2 recognition of lipoproteins contributes, however, to the total production of pro-inflammatory cytokines upon *Brucella* infection [45,46,47,48]. The initial host control of *B. abortus* depends on TLR9 recognition of *Brucella* CpG oligonucleotides, but this detection is dispensable during the chronic phase of infection [49]. TLR7 and TLR3, despite their ability to sense *B. abortus* RNA and induce proinflammatory cytokine production, do not contribute to bacterial elimination in vivo after 1 and 3 weeks post-infection [50].

In this context, it is no surprise that *Brucella* hampers TLR intracellular signalling via the production of inhibitory Toll/IL-1 receptor (TIR) domain homologs. Two homologous *Brucella* proteins (BtpA/TcpB and BtpB), translocated into host cells during infection, have been described to interfere with TLR2 and TLR4 signalling by inducing ubiquitination and degradation of the MAL/TIRAP adaptor molecule [51,52,53,54]. BtpA-mediated interference of TLR2 signalling enables *Brucella* to down-modulate the activation of infected DCs [33], whereas BtpB inhibits MyD88-mediated signalling, impacts DC maturation, and contributes to virulence in vivo [55].

*Brucella* flagellin is not recognised by TLR5 [43,56], but there is evidence of cytoplasmic detection by NLCR4, which is important for bacterial eradication in the murine model of infection [43]. This inflammatory response is, at the same time, critical for early splenic granuloma formation [43], indicating that the fine-tuning of host immunity may facilitate the establishment of a long-lasting infection. In a similar vein, in the last years, increasing evidence has emerged about the recognition of *Brucella*-derived nucleic acids by cytosolic receptors, mainly STING. This sensing leads to the ASC/inflammasome activation and is critical for host protection [57,58,59], as demonstrated for STING during the acute and chronic phases of infection in the mouse model [60]. Whether this protection operates in natural hosts is still unknown.

### 2.3. Brucella Lipopolysaccharide Determines Key Features of Bacterial Evasion and Immunomodulation

Similar to most Gram-negative bacteria, *Brucella* outer membrane contains LPS, which plays a critical function in the survival of this pathogen inside the host and in the modulation of immune responses, allowing the persistence of infection.

In contrast to the LPSs of enterobacteria, such as *Escherichia coli* or *Salmonella* spp., *Brucella* LPS displays different physicochemical properties, resulting in distinct biological traits, such as a low endotoxicity, an increased resistance to macrophage degradation, and a low immune response induction [61,62,63,64]. It differs in its whole structure from the canonical *E. coli* LPS and harbours a different O-chain, central core, and lipid A [65]. The particular O-chain of *Brucella* LPS allows bacteria (except *B. ovis* and *B. canis* that produce a rough LPS without O-chain) to enter hosts cells through interactions with lipid rafts on their surface and to inhibit lysosome fusion in murine macrophages [66]. *Brucella* O-chain also impairs complement deposition and the activation of the lectin pathway of complement [67].

The *Brucella* LPS core oligosaccharide is characterised by a low number of negative charges [68], which usually interact with polycationic peptides, C1q complement component, and the positive amino acid residues of the TLR4-MD2 receptor complex [67,69]. The branching of five sugars in the core section of *Brucella* LPS linking lipid A to the O-chain is synthesised by a mannosyltransferase, encoded by the *wadC* gene, whose disruption results in an altered core [68,69]. As such, this deletion mutant discriminates the role of the *Brucella* LPS core oligosaccharide from that of the O-chain in bacteria pathobiology. *B. abortus* Δ*wadC* is attenuated in vitro in bone marrow dendritic cells (BMDC) and macrophages (BMDM), and in vivo in a murine infection model [69]. The mutant LPS purified from a Δ*wadC* strain induces a strong proinflammatory response in a TLR-4 dependent fashion and binds to MD-2, increasing NF-κB translocation and S6 phosphorylation [69]. Concordantly, the recognition of LPS from *B. melitensis* (*Bm*)-Δ*wadC* by GM-CSF-derived (GM-)DCs and Flt3-Ligand derived (FL-)DCs triggers their maturation and a strong cytokine production, efficiently potentiating T cell proliferation in vitro [70]. Interestingly, *Bm*-wt LPS promotes the maturation of CD11b^+^ and CD24^+^ FL-cDC subsets in vitro by increasing MHC-II and costimulatory molecules expression and T helper 1 (Th1) cytokine secretion in a TLR4-dependent manner, while it poorly activates splenic cDCs subsets in vivo [70].

The impact of *Brucella* LPS extends to another relevant cell type for infection, the neutrophils. Mutant strains lacking the O-chain are more susceptible to bactericidal compounds and neutrophil degranulation [71,72]. In human neutrophils, Barquero-Calvo and collaborators have found that *Brucella* LPS is intracellularly released within vacuoles and induces premature cell death through the action of NADPH-oxidase and ROS mediators. This process distinct from necrosis, NETosis and classical apoptosis, is inflammasome-independent and associated with a low production of proinflammatory cytokines by these cells, which hampers the innate function of neutrophils [73].

### 2.4. Brucella CβG, a Cyclodextrin with a Cardinal Versatile Virulence Role

Cyclic β-1,2-d-glucans (CβG) are natural bionanopolymers present in the periplasmic space of many proteobacteria. They are cyclic molecules exclusively composed of d-glucose monomers linked by β-glycosidic bonds with, usually, 17–25 glucose units and sometimes up to 40 [74]. In several proteobacteria, the biosynthesis of CβG regulates hypo-osmotic adaptation. Nevertheless, high osmolarity does not affect the synthesis of CβG in *Brucella* by the CβG synthetase (cgs) [75], itself insensitive to elevated concentrations of solutes like KCl or potassium glutamate [76]. This cyclodextrin, however, is one of the main virulence factors of *Brucella*, as demonstrated by the significant attenuation of *B. abortus cgs* mutants both in vitro and in vivo [77,78,79].

The CβG plays a fundamental role in the intracellular survival of *Brucella* [78]. It not only modulates the lipid raft organisation by releasing cholesterol and proteins at the vacuolar membrane but also prevents the fusion of the BCV with lysosome. Indeed, interactions of the BCV with Lamp1^+^ compartments are maintained in cells infected with *cgs*-deficient *Brucella* and lost upon treatment with exogenous CβG. Thus, *Brucella* CβG controls vacuole maturation and allows intracellular bacteria to survive and reach its replicative niche, the ER [78]. In this context, the non-osmotic regulation of CβG synthesis appears highly relevant to ensure its expression before infection and all along the maturation process of the BCV, irrespective of internal solute concentrations.

Moreover, *Brucella* CβG activates human and murine DCs through mechanisms dependent on TLR4, MyD88, and TRIF, but not CD14, and promotes antigen-specific T cell responses [80]. Detailed transcriptomic analysis revealed that *Brucella* CβG triggers both pro-inflammatory and anti-inflammatory responses in DCs [81]. This oligosaccharide also drives a transient recruitment of neutrophils as compared with *Escherichia coli* LPS [81], a phenomenon that is associated with an induction of splenomegaly in mice [82]. *Brucella* CβG does not show any toxicity or immunogenicity [80], crucial features for a pathogen aiming to persist for long periods of time. The transient activation of host immune pathways in the absence of toxicity may be beneficial for the bacteria, and the recruitment of myeloid cells might profit *Brucella* for dissemination as previously suggested [1,83]. β1,2-gluco-oligosaccharides derived from *Brucella* CβG interact with the extracellular domain of DC-SIGN [84], suggesting that apart from its TLR4-dependency [80], *Brucella* CβG is recognised by the human DC-SIGN (Gorvel J.P. et al., unpublished); further investigations are nevertheless required to formally demonstrate this interaction and dissect its downstream transduction pathway.

Overall, these findings have led to the development of a recently described live-attenuated vaccine against *B. suis*, which is deficient in the phosphoglucomutase (*pgm*) gene that codes for an enzyme that catalyses the conversion of glucose-6-P to glucose-1-P, a precursor of many polysaccharides [85]. As such, this strain is incapable of producing either CβG or a complete LPS [85]. The proposed vaccine is protective in mice and unable to induce detectable levels of anti-O-antigen antibodies, an essential asset in swine vaccination campaigns for allowing discrimination between vaccinated and infected animals.

### 2.5. Brucella Host Immune System Avoidance, the Neutrophil Paradigm

The portfolio of evasion strategies displayed by *Brucella* spp. is evidenced in innate immune cells. Amongst those, neutrophils, although very well-known as short-lived phagocytic cells, contribute substantially to the persistence of infection in the long term. These cells rapidly migrate to the site of infection constituting the first line of defence; after infection, *Brucella* is opsonised and phagocytised by bovine, caprine, guinea pig, rat, canine, and human neutrophils [42,71,73,86,87,88,89,90]. The efficiency in bacterial elimination by neutrophils highly depends on the host species and *Brucella* strains [83]. Although the brucellacidal activity of granule extracts from bovine and human neutrophils has been observed after extensive contacts in the presence of myeloperoxidase, hydrogen peroxide, and potassium iodide [72], the bacteria survive inside phagocytic compartments, resisting their killing action [42,89,91] and inhibiting their degranulation [71].

The antibody-mediated depletion of neutrophils in a murine model of infection further demonstrated that these cells are not required for the early control of brucellosis [42]. Alternatively, *Brucella* directly affects PMNs’ lifespan: human neutrophils undergo premature cell death after *Brucella* infection, resulting in the exposure of phosphatidylserine on their surface without induction of a pro-inflammatory response [73]. In an in vitro murine model with opsonised-bacteria, these “eat me” signals favour the phagocytosis of infected dying neutrophils by macrophages, where the bacteria experience highly efficient replication [92], a process referred to as efferocytosis. Moreover, these macrophages are reprogrammed to an inhibitory profile secreting significant amounts of regulatory IL-10 and low quantities of TNF-α [92]. These observations support the postulated use of neutrophils as Trojan horse vehicles for the dispersion and persistence of *Brucella* [83,92], as previously reported for *Chlamydia pneumoniae* [93] and *Leishmania major* [94] infections.

Infection or exposure to *Brucella* LPS of mouse neutrophils does not induce cell death in vitro [95], a fitness that is corroborated in vivo by the absence of NETosis signs (unpublished results from our laboratory). In fact, in contrast to canine and human neutrophils, their murine counterparts fail to internalise *Brucella* when it is not opsonised, because of the exposure at their surface of N-formyl-perosamine homopolysaccharides that block recognition by opsonins until the development of adaptive immunity and the appearance of anti-*Brucella* antibodies [95]. This observation is critical when considering diverse experimental settings and also different time points analysed in the murine model. It also precludes an easy understanding of neutrophils’ biology in vivo in natural hosts. The neutrophils’ vacuolar milieu has been suggested to constitute a shelter for *Brucella* rather than a replication niche [83]. However, one must keep in mind that opsonisation seems to alter the intracellular trafficking of the bacteria by changing the nature of the BCV [96].

Intriguingly, heat-killed *B. abortus* and its lipoproteins [97] as well as *B. abortus*–infected platelets [98] can activate human neutrophils in vitro, as illustrated by enhanced oxidative burst and CD35 and CD11b expression. Although contradictory in principle, these findings suggest that (i) bacterial viability is fundamental for the aforementioned inhibition of neutrophil activation by the pathogen, and (ii) there are mechanisms that allow neutrophil detection of the infection in environments that enable their activation and may contribute to persistence through pathogenic and/or immunosuppressive mechanisms affecting adaptive immunity. In the neutropenic mutant Genista mouse model, *B. abortus* is eliminated more efficiently from the target organs, concomitantly with a significant activation of both B and T lymphocytes as well as increased levels of IFN-γ [99]. This study demonstrated for the first time that neutrophils exert a suppressive effect on Th1 responses during brucellosis in the murine model, further confirmed in a model of neutrophil depletion mediated by antibodies [100]. In the latter, the lack of neutrophils affects Th-1 responses, promotes a premature resolution of spleen inflammation and M1 macrophage polarisation [100]. The precise mechanisms by which *Brucella*-infected neutrophils regulate the adaptive response remain elusive, precluding further investigation in view of their essential role in facilitating bacterial persistence.

### 2.6. Brucella Host Immune System Avoidance, the Monocyte-Macrophage Paradigm

*Brucella* invades and replicates inside the monocytes and macrophages of different natural hosts [101] as well as those of the mouse model. Its ability to manipulate the biology of these cells is very well documented. Platelets were recently shown to promote monocyte/macrophage invasion by *B. abortus* [102]. The formation of platelet–monocyte complexes might contribute to the thrombocytopenia observed in patients with chronic brucellosis [103,104]. By hosting the bacterium hidden inside until it reaches its main replicative cellular niche, the platelet chaperones might also enhance bacterial dissemination to *Brucella*’s target organs through blood circulation and lymphatics. Indeed, lymphatic endothelial cells activate platelets, resulting in a clot that attracts monocytes by a process called lymphovenous hemostasis [105]. Consistently, bleeding complications have been reported in human brucellosis [106,107]. Another phagocytosis mode might involve outer membrane vesicles (OMV) released by *Brucella*. These OMV can be internalised by human monocytes and promote bacterial phagocytosis while inhibiting cytokine responses [108].

In mouse macrophages, large amounts of intracellular *Brucella* are not cytotoxic; this allows the bacteria to be hidden and to extensively replicate without causing any damage [42]. *Brucella*’ replication is independent of TLR2 and TLR4 signalling [42] (see above). In line with these findings, the inhibition of apoptosis in human monocytes infected with *B. suis*, which also affects non-invaded cells, indicates the participation of soluble mediators [109].

During brucellosis, macrophage and monocyte function is dysregulated. In the human host, peripheral monocytes display deficient effector phagocytic activity [110]; the proportion of CD14^hi^CD16^−^ monocytes increases [111], in contrast to the predominance of the non-classical monocyte population found in others infections, such as tuberculosis [112,113]. Infected human monocytes also present a high expression of the autophagy-related host protein LC3B, indicative of elevated autophagy levels that cause both an inhibition of the production of pro- and anti-inflammatory cytokines and an impairment of macrophage polarisation to M1 and M2 profiles in vitro [111].

Finally, *Brucella* may use monocytes as Trojan horses to cross the blood–brain barrier through the brain microvascular endothelial cells [114]. Monocytes would thus make a source of infected bacteria for other cell types within the brain parenchyma, such as microglia and astrocytes. This attractive hypothesis is based on in vitro experiments [114] and deserves further in vivo confirmation. If it holds true, it would imply a crucial role for monocytes in the pathology of neurobrucellosis, a very serious complication with deadly consequences. Additional mechanisms involving monocytes and macrophages for persistence and dissemination are summarized in the other sections of this review.

### 2.7. Brucella Impairment of Antigen Presentation and Adaptive Immunity Initiation

The various tactics detailed so far allow *Brucella* to persist inside host cells, mainly macrophages, until the initiation of adaptive immunity, when the new challenge is to confront highly effective CD4^+^ and CD8^+^ T cells. In fact, T lymphocyte responses are known to mediate protection against brucellosis [115] and are required for efficient bacterial clearance. In this regard, *Brucella* has been found to be one of the few bacterial pathogens that infect and grow inside DC [116,117]; hence, it has developed numerous stratagems to interfere with DC maturation and antigen presentation and preclude the development of an efficient immunity.

*B. suis* prevents infected human monocyte-derived DCs from inducing their maturation and secretion of TNF-α and IL-12, in a *Brucella* outer membrane protein Omp25-dependent fashion, as well as from promoting the activation of naive T cells [118]. More recently, *B. abortus* Omp25 was demonstrated to interact with the signalling lymphocytic activation molecule family member 1 (SLAMF1) expressed on the DC surface [119]. This interaction impairs DC maturation by limiting NF-κB translocation to the nucleus, which results in decreased inflammatory cytokine secretion (TNF-α, IFN-γ, and IL-6), diminished co-stimulatory molecule expression (CD80, CD86, and CD40) and T cell activation [119]. Importantly, the Omp25-SLAMF1 engagement does not affect bacterial replication during the acute phase of infection in vivo but promotes bacterial persistence at the chronic stage [119]. This study demonstrated for the first time the critical contribution of the SLAM family of receptors, described as microbial sensors in the context of *E. coli* or *Salmonella* infection of macrophages [120,121], in the initiation of anti-*Brucella* adaptive responses.

By comparing the effect on the phenotype and function of murine DCs infected by the smooth virulent *B. abortus* strain 2308 (S2308) and the rough *B. abortus* strain RB51 (licensed cattle vaccine strain), caspase-2 was found compulsory for DC maturation and the priming of T cells infected with RB51 and impaired in *B. abortus* S2308 infected-DC [122]. This observation is in concordance with previous studies, suggesting an indirect involvement of the O-side chain of the *Brucella* LPS in the maturation of human infected-DCs [117,118].

Failure of *Brucella*-induced full DC maturation has also been linked to its specific intracellular niche [40,41], the modulation of TLR signalling [33], and the singularity of its LPS [69,70]. However, some literature has described the activation/maturation of DCs upon infection [123] or stimulation with killed bacteria [124,125] or with *Brucella* proteins [48,126,127]. Such discrepancies rely on disparities in the type of stimulation, timeframe of analysis and bacterial strains used. Keeping in mind that differences exist between the *Brucella* species and also between natural, accidental, and experimental hosts is essential. For example, bovine monocyte-derived GM-DCs infected with *B. abortus* display a low expression of costimulatory molecules and cytokines but are not permissive to bacterial replication [128]. The different responses of canine and human DCs infected with *B. canis* indicate that *B. canis* induce an immune response biased towards Th1 and Th17 in canine DCs and a marked Th1 cytokine production in human DCs [129]. Finally, the viability of bacteria seems to be an important factor to appropriately stimulate DCs, as heat-killed or γ-irradiated RB51 do not induce DC maturation as well as live bacteria [124,125].

*Brucella* has evolved various strategies to overcome antigen presentation at the infection site, which might be particularly important during the chronic phase of infection when fewer bacteria are present and when avoiding T cell surveillance of infected cells becomes critical. Early studies have shown that *B. abortus* LPS accumulates in lysosomes of infected macrophages and that, later on, it reaches the membrane shaped in large clusters with the O-chain facing the extracellular milieu [130,131]. These macrodomains are enriched with MHC-II molecules, thus interfering with this presentation pathway and inhibiting the capacity of macrophages to present antigenic peptides to CD4^+^ T cells [62]. A similar interaction has been observed in murine and human B-cell lines, which might affect the anti-LPS humoral immune response [132].

In addition, Barrionuevo et al. demonstrated that *Brucella* infection decreases the expression of MHC-II in IFN-γ-stimulated macrophages and, therefore, abolishes antigen presentation and CD4^+^ T cell recognition of infected cells [47]. This inhibition of MHC-II expression is mediated by the TLR2 signalling and IL-6 secretion and induced by the recognition of the *Brucella* outer membrane lipoprotein Omp19 [47,133]. The IL-6-dependent MHC class II downregulation is driven by an inhibition of IRF-1 expression, which decreases the expression of CIITA, a master regulator of MHC-II, and is mediated by *B. abortus* and its lipoproteins [134].

The impact of *Brucella* infection on macrophage antigen presentation also involves the inhibition of MHC-I expression and results in diminished CD8^+^ cytotoxic T cell responses [135]. By exploiting the EGFR-ERK signalling pathway [136], a *Brucella* infection of IFN-γ-stimulated macrophages does not induce changes in protein synthesis but causes MHC-I molecule retention within the Golgi apparatus [135]. Bacterial RNA is the structural component responsible for such MHC-I downregulation in human monocytes in a TLR-8-dependent manner [137]. Thus, *Brucella* viability and its PAMP RNA and RNA degradation products seem to be essential for the avoidance of cytotoxic CD8^+^ T cell immunological surveillance.

**Figure 1:**
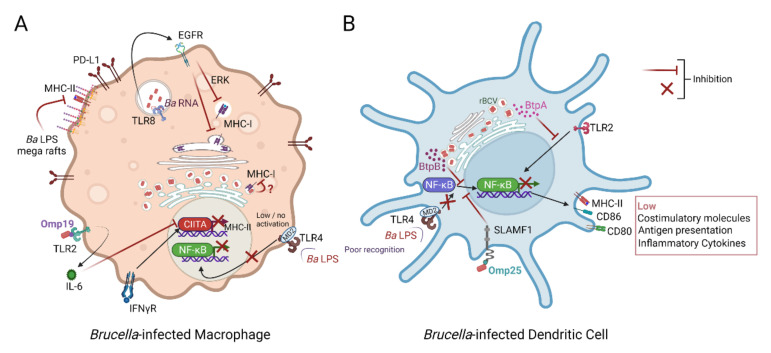
**Mechanisms of *Brucella’s* antigen presentation impairment**. *Brucella* has evolved multiple ways to dampen antigen presentation by (**A**) macrophages and (**B**) dendritic cells, preventing proficient development of an adaptive immunity. (**A**) Recognition of *Brucella* lipoproteins by TLR2 leads to IL-6-dependent inhibition of the transcription factor CIITA, resulting in diminished transcription and expression of IFN-γ-induced MHC-II. Moreover, *Brucella abortus* LPS (*Ba* LPS) reaches the cell surface, forming macrodomains with MHC-II molecules and interfering with the presentation of peptides to CD4^+^ T cells. This impairment also influences cytotoxic CD8^+^ T cells, since recognition of *Brucella abortus* RNA (*Ba* RNA) induces retention of MHCI molecules within the Golgi apparatus via TLR8 and the EGFR pathway. (**B**) The Omp25-SLAMF1 interaction limits NF-κB translocation to the nucleus, decreasing pro-inflammatory cytokine secretion and costimulatory molecules expression in dendritic cells. The *Brucella* effectors BtpA and BtpB, translocated to the cytoplasm during infection, interfere with TLR2 and TLR4 signalling and control dendritic cell maturation. In both cell types, the peculiar structure of the *Ba* LPS, specially its core, makes it poorly recognised by the TLR4-MD2 complex preventing full activation, NF-κB translocation and impairing dendritic cell maturation and T cell activation. Nuclear phosphorylated active NF-κB dimers are represented in green.

In conclusion, there is vast evidence for the manipulation of antigen presentation mechanisms to impair host immunity and favour brucellosis chronicity, as illustrated in Figure 1. It is of note that inhibition is only partial, as demonstrated by the induction of protective Th1 responses; the fine regulation mediated by the dysregulated expression of co-stimulatory molecules and cytokines in the microenvironment may result, at the same time, in not fully effective responses or negative regulators thereof.

### 2.8. Brucella Modulation of T Lymphocyte Responses

The direct and indirect effects of *Brucella* on antigen-presenting cells described above necessarily impact on the development of the adaptive immune response against the bacteria. It is well-known that Th1 immune responses and the production of interferon gamma (IFN-γ) are crucial for the control of brucellosis [138,139,140]. In fact, by using a panel of genetically deficient mice, a key role for IFN-γ-producer CD4^+^ T cells in restraining *B. melitensis* primary infection was unravelled, in contrast to the modest contribution of CD8^+^ T cells and B cell-mediated responses [141]. Interestingly, chronic brucellosis patients present diminished proportion of Th1 lymphocytes as compared with acute brucellosis patients [142,143]; peripheral blood mononuclear cells (PBMCs) from chronic patients also secrete less IFN-γ in response to *Brucella* antigen stimulation [144].

In the first days of infection, the VirB operon contributes to the Th1 polarisation of CD4^+^ T cells in *B. abortus*-infected mice [145]. At chronic stages of *B. melitensis* infection in mice, CD209^+^ marginal zone (MZ) macrophages and CD169^+^ marginal metallophilic macrophages are decreased in the spleen [146]. Remarkably, this cell loss does not occur in IFN-γR^−/−^ mice, suggesting that *Brucella* infection alters MZ macrophage populations through a sustained low-level of IFN-γ signalling, which in turn would reduce the ability of the spleen to deliver antigen and control systemic infection [146].

In the establishment of chronic brucellosis, the development of a proficient Th1 response seems to be impaired by several mechanisms, including the expansion of regulatory T cells with immunosuppressive capacity [147,148]. The percentages of regulatory lymphocytes (Tregs, defined as CD4^+^CD25^+^FoxP3^+^ T cells) are significantly elevated in the peripheral blood from both acute and chronic brucellosis patients [149,150,151] and restored to healthy control levels after regular antibiotic treatment [150]. In the mouse model, Tregs exert suppressive functions regulating effector T cell proliferation and IFN-γ production in *B. abortus*-infected BALB/c mice; consistently, antibody-mediated depletion of this population reduces bacterial colonisation [147]. Tregs expand and promote the persistence of *B. abortus* not only in the spleen but also in the uterus of infected mice, and their inactivation with tumour necrosis factor receptor II (TNFR2) antagonistic antibody significantly decreases the bacterial burden [148]. This means that this subpopulation of T cells plays an undeniable role in uterine tropism and the chronicity of brucellosis.

The direct suppression of T effector cells by Tregs may involve (i) immunosuppressive soluble factors or (ii) cell contact. As regards the first mechanism, elevated TGF-β plasmatic levels have been reported in brucellosis patients as compared to that of healthy controls [144,152], which correlate with diminished lymphoproliferative responses to *Brucella* antigens [152]. Treatment of PBMC with an anti-TGF-β neutralizing antibody restores T cell proliferation [152], demonstrating the suppressive function of TGF-β. Moreover, genetic associative studies identified single nucleotide polymorphisms linked to brucellosis susceptibility in the *TGFB* gene [153,154]. IL-10, another cytokine well-known for its ability to inhibit the development of Th1 type responses [155], is also induced during *B. abortus* infection in the mouse model [140,156,157] and increased in serum from acute and chronic patients [158,159]. IL-10^−/−^ mice produce higher levels of IFN-γ and better eliminate *B. abortus* than WT animals [160]; in addition, by modulating macrophage functions, IL-10 contributes to create an environment that allows enhanced bacterial survival and persistent infection [161].

The second immunosuppressive mechanism comprises the action of immune checkpoints and inhibitory receptors, extensively investigated in the context of cancer, albeit more recently during viral and bacterial infections [162,163,164,165]. Their contribution to brucellosis pathogenesis and persistence remains, so far, an open field. Frequencies of circulating GITR^+^ and PD-1^+^ Tregs are elevated in both acute and chronic brucellosis patients in comparison to those of healthy individuals, whereas CTLA-4^+^ Tregs are significantly increased in chronic patients only [159]. PD-1 expression is also augmented in human CD8^+^ T cells [142], in agreement with a subset of *Brucella*-responsive CD8^+^ T cells found in chronically infected mice, which are capable of inhibiting IFN-γ production and delaying memory responses [166]. Given the importance of these immunoreceptors in dampening the host immune response and the development of several blocking antibodies against the PD-1/PD-L1 pathway for cancer therapy available for use in humans, the study of the mechanisms of action of these receptors in the immune cells during brucellosis deserves further investigation. Unpublished data from the Gorvel lab pointed out a selective upregulation of the *PDL1* gene together with other immunosuppressive marker genes in whole blood of acutely infected patients, confirmed at the protein level all along the course of infection in the murine model of brucellosis (Gonzalez-Espinoza et al., 2022, in preparation).

The response mediated by CD8^+^ T cells also contributes to the protection against *Brucella* via the secretion of IFN-γ, even though their main function is to eliminate infected cells by Fas–FasL interaction and/or the secretion of perforins and granzymes [167,168,169]. During *B. melitensis* chronic infection of mice, CD8^+^ T cells characterised by an exhausted phenotype (PD-1^+^RAG-1^+^) and lacking polyfunctional cytokine production [168] are found in the spleen. Their suppressed phenotype stands apart from the other exhausted CD8^+^ T cells, as they express IFN-γ only [168]; it is most probably related to environmental cues. There is evidence that these *Brucella*-exhausted CD8^+^ cells can recover function after virulent transfer to a new host milieu [166]. In addition, the pathogenic *B. melitensis* protein BtpA/TcpB directly contributes to the evasion of cytotoxic responses by blocking the CD8^+^ T cell killing of specific targets [168]. The cytotoxic potential of a CD4^+^ T cell population against *B. abortus* infection in mice has also been disclosed [170], but further studies are needed to unravel the exact contribution of this population during the chronic stage of infection.

Finally, other described strategies that *Brucella* uses to impair T-cell mediated responses comprise the TNF-α-dependent induction of apoptosis in human T cells [171], the inhibition of CD4^+^ T cell-mediated immunity by B cells through an enhanced MHC-II-dependent production of IL-10 by T helper cells [172], and the suppressive effects of neutrophils on Th1 responses during *B. abortus* infection [99].

### 2.9. Brucella Modulation of Immunometabolism

Lately, there has been a tremendous interest in the understanding of the intricate metabolic network governing the immune cells’ functionality and behaviour, which might provide attractive therapeutic targets not only for cancer and autoimmune disorders but also for infectious diseases. Several studies have now described tactics used by intracellular pathogens to manipulate host immunometabolism and establish a chronic infection.

As regards brucellosis, in infected macrophages, the transition from nitric oxide production to the biosynthesis of polyamines promotes both the intracellular survival of *B. abortus* and chronic infection in mice [173]. In addition, *Brucella* infection disrupts mitochondrial function and localization, and completely affects the metabolism of all of the amino acids known to enter the tricarboxylic acid cycle [174]. Thus, *Brucella* adapts its metabolic requirements to meet the needs of its preferential cellular niche, the non-inflammatory macrophages, during the chronic phase of infection [175].

The peroxisome proliferator-activated receptor γ (PPARγ) pathway is increased during the chronic stage of infection in the splenic myeloid cells of *B. abortus*-infected mice, eliciting a rise in intracellular glucose availability within the cell [176]. This PPARγ-mediated shift from oxidative metabolism of glucose to β-oxidation of fatty acids is predominant in alternatively activated or M2-like macrophages, the prevailing macrophages at this stage [176]. The elevated intracellular glucose availability allows *Brucella* to preferentially replicate inside these cells through a mechanism dependent on the ability of *B. abortus* to uptake glucose via its transporter gluP [176]. Likewise, PPARγ is a critical regulator of the metabolic environment set up essential for long-term *Salmonella* persistence [177]. MyD88-dependent changes in the host metabolism also contribute to the control of *Brucella* infection in mouse models by regulating glucose availability as well as lactate production, the latter bearing recognised antibacterial effects [178]. *B. abortus* efficiently metabolises lactate as a carbon source inside human THP-1 cells, and lactate is essential for the intracellular replication of the bacterium; hence, *Brucella* takes direct advantage of this Warburg-like shift in host inflammatory cells to support its in vitro growth in macrophages during infection [174].

Some *Brucella* effectors directly modulate the host metabolism. For example, BtpA and BtpB regulate energy metabolism through NAD^+^ hydrolysis in HeLa cells and immortalised bone marrow-derived macrophages [179]. NAD^+^/NADH is tightly linked to serine catabolism, recently reported to be important for *Brucella* intracellular proliferation and pathogenesis [180]. Interestingly, serine is considered a key immune metabolite that directly shapes adaptive immunity by controlling T cell proliferation [181]. Most of the findings herein described are focused on the bacterial modulation of the host metabolism in infected macrophages, the favoured cellular niche of *Brucella*, but the interactions with other cell types (i.e., neutrophils, lymphocytes among others) in terms of immunometabolism regulation remain uncharacterised and deserve investigation.

Collectively, it is increasingly evident that the complex host metabolism network plays a crucial role during intracellular infections, and that immunometabolic regulators become attractive targets with promising translational avenues. The manipulation of metabolic hubs might help not only to reduce available nutrients for *Brucella* but also to overcome immune cell reprogramming for treating brucellosis [175].

## 3. Current Knowledge of Immunosuppression and Chronicity through Other Intracellular Bacteria

In the field of intracellular bacterial infections leading to chronic diseases, pathogens seem to share common strategies to persist in the host. This is determined not only by the bacteria’s ability to evade microbicidal mechanisms but, importantly, to use immunity for their own benefit. This section will focus on infectious diseases, including brucellosis as well as tuberculosis, salmonellosis, and chlamydiosis, among others, which are worldwide health concerns.

### 3.1. Granulomas and “the Wall Effect” in Bacterial Infections

Granulomas are structured aggregates of macrophages, of various types with specific morphologies, accompanied with other immune cells at the infection site [182]. Inside these heterogeneous bodies, bacteria constantly escape from the dormant state induced by the low-oxygen environment and replenish the volume of replicative bacilli, thus remaining viable for decades.

*Brucella* triggers the formation of granulomas in the spleen and liver from natural hosts, humans, and mice [183,184,185,186]. These structures are enriched in F4/80^+^MHC-II^+^CD11b^+^ activated macrophages, with *Brucella* infection being restricted to macrophages, monocytes, and DCs, but not granulocytes and hepatocytes [186]. The proper development of *Brucella*-containing granuloma depends on MyD88, Nod-like receptor (NLRP)12, IL-12, and IFN-γ [186,187]. Remarkably, NLRP12 negatively regulates MAPK signalling and NF-κB activation, thus leading to reduced IL-12 and IL-1β secretion upon *Brucella* infection. As such, a tight control of these Th1 cytokine levels seems to be required for the proper development of *Brucella* granulomas. Despite these observations and the suggestion of granulomas as helpers for persistent infection, the precise role of these structures during *Brucella* infection remains to be clarified, as well as the mechanisms that govern their formation, dynamics, and the relationship with anti-*Brucella* adaptive immunity.

The formation of granuloma is a remarkable hallmark of tuberculosis (TB), an infectious disease transmitted by the bacterium Mycobacterium tuberculosis, primarily affecting the lungs, and one of the top ten causes of death from a single infectious agent [188]. The TB granuloma is characterised by a very well-organized and heterogeneous structure, composed of a diversity of immune cell types. It contains a central core made of M. tuberculosis-infected and uninfected macrophages (with or without a region of caseous necrosis), epithelioid macrophages, and multinucleated giant cells (also known as Langhans giant cells) [189,190]. This core is surrounded by a cuff of B, α/β, and γ/δ T lymphocytes, and associated tertiary lymphoid structures. Other cell populations recruited to the granuloma include neutrophils, DCs, eosinophils, mast cells, innate lymphoid cells (ILCs), and also non-hematopoietic cells, such as fibroblast and endothelial cells [191,192,193].

Traditionally, the TB granuloma has been considered to be beneficial for the host by forming a physical barrier that prevents bacterial escape. Nevertheless, there is a growing awareness that it also provides a permissive environment for M. tuberculosis, in part by limiting the positioning, survival, and function of T cells [194,195]. In fact, although bacterial growth and dissemination are restricted via the production of pro-inflammatory cytokines, such as IFN-γ, TNF-α, IL-17 and the presence of an oxygen-limited environment, the access of immune effectors to the site of infection is still debated. The granuloma core is enriched for anti-inflammatory mediators (like TGF-β, IL-10 [196], prostaglandins [197]) and inhibitory receptors (such as PD-L1, specifically in myeloid cells and proliferating regulatory T cells [198,199]) that contribute to impairing efficient lymphocyte responses. T lymphocytes surrounding the granuloma core express higher levels of TIM-3, CTLA-4, and LAG-3 [195,200,201,202,203,204], molecules usually associated with an exhausted and inhibitory phenotype.

Historically, there has been a tendency to consider granulomas as stable structures where the bacilli remain enclosed. Nowadays, the “dynamic hypothesis” has gained ground, based on intravital imaging, which revealed a constant movement of cells in and out of the granuloma, including cells with latent bacilli that are drained into the bronchioles, opening up the possibility of continuous reinfections [205].

Beside brucellosis, TB is not the only infectious disease to develop granulomas. Other intracellular bacteria known to develop this kind of structures are *Coxiella burnetti* (etiologic agent of the Q-fever) [206], *Bartonella henselae* (Cat-scratch disease) [207], *Tropheryma whipplei* (Whipple’s Disease) [208], *Actinomyces israelii* (actinomycosis) [209], and *Salmonella enterica* Typhimurium (gastroenteritis) [210], among others.

*Salmonella enterica* are Gram-negative intracellular bacteria that infect humans and animals and are a major cause of food-borne illness. Ranging from self-limiting gastroenteritis to lethal bacteremia, salmonellosis annually causes up to 1.3 billion new cases around the globe of non-typhoidal *Salmonella* infections [211] and 20 million cases of enteric fever from typhoidal *S. enterica* serovars [212]. After oral ingestion, *Salmonella* infects the intestinal tract and disseminates to cause systemic infection of organs, including liver and spleen [211]. The regulation and dynamics of *Salmonella*-induced splenic granuloma composition has been analysed in-depth these last years. At 4 weeks post-infection, granulomas become more confluent, with macrophages occasionally harbouring outstretched morphology as well as infiltrated neutrophils [213]. At day 42 post-inoculation, *Salmonella enterica* Typhimurium survives inside iNOS^+^ monocyte-derived macrophages in splenic granulomas regardless of the Th1 response [210]. The bacteria are indeed largely inaccessible to CD4^+^ T cells, thanks to a local CXCL9/10 production by monocytes that induces the positioning of T lymphocytes in restricted peripheral areas [210]. At 2–3 months post-infection, splenic granulomas comprise cohesive aggregates of macrophages with external intermittent margins of lymphocytes and even fewer mingled neutrophils. These macrophages present a heterogeneous phenotype, with different types of M2 polarised cells: the IL4R-α^+^ that host the niche of intracellular *Salmonella* and the CD301^+^ that provide a favourable environment for persistence. TNF-α signalling controls granuloma macrophage M2 polarisation, partly mediated by the *Salmonella* effector SteE that interacts with Stat3, thus favouring *Salmonella* maintenance [213]. These findings are in concordance with the formation of granulomas in the liver of *Salmonella*-infected mice [214] and with the anatomically beneficial environment of the hepatobiliary system that may contribute to the persistence for extended periods of infection (more than two years) [215].

The extensive knowledge of granuloma formation and composition gained in the TB and *Salmonella* infection models has led to the identification of a coexistence of various M1 and M2 macrophages inside granulomae, which probably holds true for other intracellular bacteria. Hence, it suggests that manipulating the polarisation of host macrophages by intracellular bacteria may be a way to subdue the host control of pathogen persistence. It also supports M2 polarisation as a possible therapeutic target for chronic stages of infection.

### 3.2. Pyroptosis, a Cellular Death Process Co-Opted by Bacteria

Pyropotosis is a Caspase-1/IL-1β-mediated form of programmed cell death that involves gasdermin family members and is associated with pore-induced lysis [216].

Several components of the signalling cascade leading to pyroptosis are activated in brucellosis. The host protein AIM2 senses *Brucella* DNA to activate the NLRP3 inflammasome, a complex essential for the secretion of caspase-1-mediated IL-1β and for the resistance of mice to *Brucella* infection [217]. NLRP3 activation by *Brucella* depends partially on the mitochondrial generation of ROS. The ER stress caused by *Brucella* infection is sensed by the UPR via IRE1 and TXNIP and thus linked to mitochondrial ROS and inflammasome assembly [218]. Although non-canonical inflammasome activation has been attributed to gasdermin-D-mediated cell death triggered by *B. abortus* [58], there is a decreased bacterial load in the absence of Caspase-11 and a significantly greater role for the canonical ASC inflammasome in the host defence against this pathogen. Pyroptosis induction is attributed to the recognition of bacterial gDNA in the cytosol [58,59]. Interestingly, the lack of ASC improves bacterial clearance from infected BMDMs in vitro [59], opening up the possibility of the subversion of this process by *Brucella* for its own benefit. One putative mechanism might involve the activity of the effector protein TcpB, which promotes ubiquitination and degradation of caspases 1, 4, and 11, thus attenuating *Brucella*-induced pyroptosis and secretion of inflammatory cytokines [219].

Following internalisation, *Salmonella* establishes its intracellular niche in a modified phagosome, known as the *Salmonella*-containing vacuole (SCV), managing to survive and proliferate. Wild-type (WT) *Salmonella* damages its nascent vacuole and reaches the cytosol of epithelial cells and macrophages, where it eventually hyper-replicates [220,221]. This presence in the cytosol proceeds with the recognition by several host sensors and activation of canonical and non-canonical inflammasomes, according to the disease stage and cell type [222]. During infection at the gastrointestinal mucosa, bacteria express the pathogenicity island-1 of the type III secretion system (T3SS-1) and flagellin. This concerted expression triggers assembly of the NAIP/NLRC4 inflammasome in infected epithelial cells, which mediates IL-1 family cytokine secretion and pyroptosis [223,224,225,226,227]. In consequence, epithelial cells are expelled into the lumen, thus limiting the pathogen’s intraepithelial proliferation [223,224,226]. Conversely, NAIP/NLRC4 in phagocytes is dispensable for the restriction of *Salmonella* dissemination and replication at infection sites during early infection [223]. In this case, the defence ability of epithelial cells in vivo is supported by a specific high NAIP/NLRC4 expression and the necessity for epithelium-invading *Salmonella* to express the NAIP ligands-flagella and T3SS-1 [223].

However, by using counter-regulatory mechanisms that induce the repression of T3SS-1 and flagellin expressions [223,228], *Salmonella* manages to evade this intrinsic host defence and horizontally invade nearby cells, ultimately replicating within macrophages to facilitate systemic infection and chronic colonisation [229,230]. Later in the systemic phase, a delayed form of caspase-1-dependent pyroptosis is activated, which requires the pathogenicity island-2 of the T3SS (T3SS-2) and the *spv* genes [231,232]. This process might require recognition of different bacterial components, such as LPS and DNA, and is mediated by the protein kinase PKR after TLR4 activation [233]. *Salmonella* also uses the T3SS-2 to damage the SCV inducing cell death and a complement-dependent “eat-me” signal for neutrophil efferocytosis [234]. Importantly, bacteria entrapped in these efferocytosed cells are protected from the killing action of neutrophils, in contrast with bacteria internalised by phagocytosis [234]. Consequently, this virulence factor-mediated mechanism enables bacteria to evade host control during systemic infection and contributes to persistence.

In view of the model of *Salmonella* infection, where different consequences of pyroptosis cell death have been described depending on the cell type and stage of infection, it is critical to delve into the chronic stages of brucellosis and specific sites of the infection. Many questions remain to be answered: What is the ability of *Brucella* to induce pyroptosis in different foci of infection? What is the consequence for the pathogenesis and persistence of infection? What host and bacterial factors are involved? What role does neutrophil efferocytosis play after macrophage pyroptosis in vivo? When mimicking the chronic phase of infection by using WT opsonised *B. abortus*, the type 4 secretion system (T4SS) perforates the BCV, allowing complement penetration, a process that triggers efferocytosis by neutrophils [234]. This might lead, as demonstrated for *Salmonella*, to protecting the bacterium engulfed in pore-induced intracellular traps from the respiratory burst despite ROS production and to promote the survival of *Brucella* inside neutrophils [234].

### 3.3. Type I and III IFNs in the Context of Persistent Bacterial Infections

Type I interferons (IFNs) form a family of widely expressed inducible cytokines whose most abundant and known members are IFN-α and IFN-β. They have been identified more than sixty years ago for their antiviral response and, more recently, have been reported to be involved in bacterial responses but with disparate functions, including an anti-inflammatory one [235]. Type I IFNs signal through the same common heterodimer α/β receptor (IFNAR) to induce the expression of more than 300 stimulated genes (ISGs). These cytokines are produced by almost all cells in the body in response to the activation of PRRs by microbial products. Type I IFNs are very well known for their capacity to trigger an antiviral response by inducing effector molecules encoded by ISGs that interfere with viral replication through various mechanisms [236,237]. However, the role of type I IFNs during bacterial infections is highly context-dependent with both beneficial and detrimental outcomes for the host.

As regards brucellosis, cytosolic *Brucella* DNA is sensed by STING, which is required for IRF3-mediated type I IFN production in infected murine macrophages [238]. *Brucella* infection drives a modest IFN-β induction and type I IFN transcriptional program in GM-CSF-derived human DCs in vitro [33,41]. However, IFNAR-deficient mice present a reduced bacterial burden compared to that of WT mice, most probably due in part to the elevated IFN-γ and NO production by IFNAR^−/−^ splenocytes and their diminished apoptosis [238]. These findings demonstrate that type I IFN signalling induced by *B. abortus* plays an anti-inflammatory role and is tightly controlled by the pathogen. The mechanisms involved remain unexplored but are expected to be central modulators of host–pathogen interactions. What are the effects of type I IFN signalling on each cell population; what is the synergy/interaction with IFN-γ and Th1 responses, and what are the main sources of type I IFNs during *Brucella* infection are key questions looking for answers. For instance, plasmacytoid DCs are predominant type I IFN producers and are very well known for their anti-viral responses, but their role during bacterial infections has been much less studied. In this context, bats have been reported as important reservoirs of zoonotic viruses with a great ability to tolerate infections potentially lethal in other hosts [239]. Given their highly resistant immune system, bats emerge as an interesting model to study type I IFNs during bacterial zoonotic infections.

The most common bacterial sexually transmitted infection is caused by *Chlamydia trachomatis* [240], an intracellular Gram-negative bacterium that replicates only inside a vacuole termed inclusion body, primarily in mucosal epithelial cells. Like *Brucella*, *Chlamydia* infection is mostly asymptomatic, consequently often undiagnosed and untreated; but during chronic stages, it leads to pelvic inflammatory disease, infertility, ectopic pregnancy, and chronic pelvic pain in women [241] and urethritis and epididymitis in men [242]. Upon *Chlamydia trachomatis* infection, there is a strong induction of type I IFN production by multiple cell types, including macrophages [243] and oviduct epithelial cells [244]. The induction of IFN-β results from the sensing of cytosolic DNA by cyclic GMP-AMP synthase (cGAS) and the later activation of STING [245]. Type I IFN synthesis in oviduct epithelial cells occurs in two waves, an early one that is TLR-3-dependent and involves IRF3 and a late stage one, triggered by soluble factors and requiring IRF7 [244].

Initial studies have shown that in vitro treatment with type I IFN contributes to decreasing bacterial load, possibly through the depletion of intracellular iron and induction of L-tryptophan catabolism via the indoleamine 2,3-dioxygenase (IDO) [246,247]. However, at late stages of a murine model of genital infection with *Chlamydia muridarum*, type I IFN has a detrimental role, as IFNAR^−/−^ mice exhibit a more rapid resolution of infection and less pathology than WT mice. This might be explained by increased *Chlamydia*-specific CD4^+^ T cell responses in the absence of type I IFN signalling [248]. Accordingly, type I IFN negatively regulates CD8^+^ T cell responses [249] and also enhances regulatory T cell induction through Foxp3 acetylation [249]. Similar susceptibility to *Listeria monocytogenes* infection was observed in IFNAR^−/−^ mice, together with a decreased splenic apoptosis [250], while increased resistance to intradermal infection with *Francisella tularensis* (the etiologic agent of tularemia) in these deficient mice was associated with an expansion of IL-17+ γ/δ T cells and neutrophils [251].

Patients with active TB display a type I IFN-inducible blood transcriptional signature [252,253,254], associated with an extended lung radiographic disease and diminished treatment success rates [252]. Likewise, elevated production of type I IFN has been linked to the virulence of *Mtb* strains and increased host susceptibility [255,256,257,258]. The mechanisms behind this exacerbation of *Mtb* infection remain not fully understood. They include enhanced IL-10 production, the suppression of protective cytokines [258,259,260,261], the inhibition of myeloid cell responsiveness to IFN-γ [262], and the promotion of alveolar macrophages cell death [263], among others and have been extensively reviewed [264].

Finally, type III IFNs, also known as IFN-λ, deserve special attention in light of recent discoveries highlighting their capacity to promote milder inflammatory responses compared with type I IFNs [265,266] and to shape mucosal barriers integrity and function [267]. *Listeria monocytogenes* was the first bacteria shown to induce type III IFNs, specifically by epithelial cells, hepatocytes, and placental throphoblasts [268,269]. Such induction was later observed during infections with *Borrelia burgdorferi* [270], *M. tuberculosis* [271], *Pseudomonas aeruginosa* [272,273], *Staphylococcus aureus* [269,274], and *Salmonella* Typhimurium [269] among others. The physiological significance of the type III IFN pathway in most of these infections is yet to be determined. To date, there is only one study reporting increased IL-29 (IFN-λ1) and IL-28A (IFN-λ2) serum levels in acute brucellosis patients as compared with patients after standard anti-brucellosis treatment [275], without any assumption regarding the putative role of these type III IFNs during the acute or chronic phase of brucellosis.

### 3.4. Intracellular Bacteria and B Cells, an Overlooked Association

The importance of humoral immunity and B cell responses in intracellular infections has been commonly neglected; however, emerging evidence reveals that they are relevant pieces of the host immunity puzzle.

The protection conferred by antibodies during infection with *Brucella* has been little studied so far, although their detection for serological diagnostic use after infection or vaccination in animals and humans has been better exploited [276]. The administration of monoclonal antibodies specific for the O-chain of *Brucella* LPS demonstrated a certain level of protection against *Brucella abortus* infection in mice [277]. *Brucella* infection elicits the production of high titers of IgG and IgA antibodies, that gradually fall off after treatment in most cases [278]. High titers of immunoglobulins characterise relapse or chronic phase of the disease [278]. However, there is a lack of correlation between anti-*Brucella* antibodies and clinical outcomes and culture positivity [279]. Moreover, as discussed in previous sections, the intracellular fate of the bacterium differs whether it is opsonised or not, highlighting the role of the humoral response.

*B. melitensis*, *B. abortus*, *B. ovis*, *B. canis*, and *B. suis* are able to infect human B lymphocytes [280]. B-cell deficient infected mice display higher resistance to *Brucella* infection, together with an enhanced frequency of IFN-γ-producing CD4^+^ and CD8^+^ T cells and decreased IL-10-producing cells [281]. Marginal Zone B cells are more permissive to *Brucella* infection than follicular B cells, and B lymphocytes secrete TGF-β and IL-10 during the early stages of infection in WT mice [281]. Concordantly, by using B cell-deficient mice and adoptive transfer experiments, B cells were shown to inhibit CD4^+^ T cell-mediated immunity against *B. melitensis* in a MHCII-dependent manner [172]. Mechanistically, the *B. abortus* virulence factor PrpA stimulates B lymphocyte polyclonal activation and IL-10 production and is required for establishing a successful chronic infection [282]. Altogether, these findings suggest that *Brucella* co-opts B cell biology in order to (i) persist during long periods of time in an unperturbed reservoir and (ii) modulate the host immune response for its own benefit. However, as compared to other infections, there is a long way to go to a better understanding the bacterial factors involved, the relevance of the crosstalk with other cell types, and the impact on their function. The exact contribution of antibodies in this scenario remains undetermined.

In the last decade, clinical studies have pointed out that the lack of *Salmonella*-specific antibodies at young age correlates with the incidence of nontyphoidal salmonellosis in African children [283], even in the presence of *Salmonella*-specific CD4^+^ T cells [284]. Conversely, the adoptive transfer of antibodies without T cell-mediated immunity does not confer protection against *Salmonella* infection [285].

The B cell response to *Salmonella enterica* Typhimurium occurs massively at extrafollicular sites, where switched antibody response happens, while germinal centres formation is greatly delayed [286,287]. Bacterial LPS, outer membrane proteins and flagellin have been identified as the main target antigens of the antibody response [288,289,290,291]. A recurrent question that arises when studying humoral responses in these infections is how (and when) an intracellular bacteria can be targeted by antibodies. In this regard, there is evidence that some bacteria can be found as extracellular bacteria in the bloodstream [292,293,294], in addition to other bacilli that escape the lesions [295] and therefore become accessible to antibodies. Then, the known mechanisms of action involving anti-*Salmonella* antibodies include either opsonisation (enhance phagocytosis targeting the bacteria to immune receptors) [296,297] or the activation of the classical complement pathway [283,298], which leads to bacterial elimination, but also the recognition and activation of monocytes, macrophages and possibly other cell types, such as NK cells and neutrophils, through Fc-receptor engagement [299,300,301,302].

In this context, *Salmonella* has evolved strategies to impair humoral responses, facilitating its spread and persistence. For instance, during *Salmonella enterica* Typhimurium infection, IL-12 suppresses T follicular helper differentiation and therefore inhibits germinal centre formation and influences affinity maturation and long-lived humoral immunity [303]. Chronic antigen stimulation during *Salmonella* infection causes the mobilization of long-lived plasma cells from the bone marrow via TNF-α, with an associated decrease in circulating antibody levels [304]. Furthermore, the *Salmonella* protein SiiE specifically diminishes the number of IgG-secreting plasma cells in the bone marrow, contributing to a reduction in IgG titers in the serum [305].

B cell biology is not limited to antibody production; B cells exert also important functions as antigen-presenting and regulatory cells. It is well established that B cells are targets of intracellular bacteria, including *Brucella*, *Salmonella*, *M. tuberculosis*, and *Francisella tularensis*, among others [306]. In contrast to its effect on epithelial cells and macrophages, *Salmonella* inhibits pyroptosis in murine splenic B cells and abrogates IL-1β production by impairing NLRC4 transcription. This allows *Salmonella* to survive in these cells for long periods of time [307,308]. Moreover, MyD88 signalling is critical to promoting IL-10 production in *Salmonella*-infected B cells, restraining the activity of neutrophils, NK cells, and inflammatory T cells and preventing bacterial clearance [309]. IL-10 production has been postulated as one of the main suppressive mechanisms by regulatory B cells (Breg). Consistently, an increased proportion of CD19^+^CD5^+^CD1d^+^ Bregs has been observed in the peripheral blood of patients with active tuberculosis [310], concomitant with the suppression of Th17 responses and the inhibition of IL-22 production [310]. B-reg-mediated weak protective T cell responses also operate during *Chlamydia muridarum* genital tract infection [311,312]. Finally, *Salmonella*-infected murine B cells express PD-L1 and PD-L2 [163], whose interactions with PD-1 contribute to the impairment of CD8+ T cell responses [313].

These various examples illustrate the wide role played by B cells during intracellular bacterial infections that beside the classical antibody production and its subversion is only emerging.

### 3.5. Innate Lymphoid Cells: Novel Innate Players in Bacterial Infections

Other cells that have been understudied in the field of bacterial infections are the ILCs, which correspond to the innate counterparts of T lymphocytes lacking genetically recombined adaptive antigen receptors. They form an heterogeneous group of potent innate effector cells, known by their tissue-resident sentinel functions, which have been recently reclassified into five subsets based on their development and function [314]. These subsets comprise natural killer (NK) cells (mirroring the functions of cytotoxic CD8^+^ T cells), ILC1s, ILC2s, ILC3s (that mirror CD4^+^ Th1, Th2, and Th17 cells, respectively), and lymphoid tissue-inducer cells (LTi, critical for the development of lymph nodes and Peyer’s patches during embryogenesis) [315,316]. During the last decade, some groups have shed light on the induction and function of these cell subsets during bacterial infections, although there is still a long way to go before their role and the way their biology is manipulated by pathogens is fully understood.

Upon *B. melitensis* infection, Lacey et al. uncovered that ILCs limit *Brucella*-induced joint swelling and participate to local IFN-γ production [317]. In turn, IFN-γ-dependent Nitric Oxide contributes to inhibiting inflammasome activation and suppressing bacterial-induced arthritis [317]. Human NK cells in vitro impair intracellular *B. suis* multiplication through the activation and induction of NK cell cytotoxicity against infected macrophages [318]. However, no significant role of these cells has been found in the early control of *Brucella abortus* in vivo infection when using antibody-mediated depletion approaches [319]. Thus, it was hypothesized that *Brucella* might avoid NK cell control by harming the activity of these cells in the acute phase of infection. Accordingly, clinical observations show an impaired functionality of NK cells in patients with acute brucellosis [320].

During *M. tuberculosis* infection, ILCs become activated and accumulate in the lungs [321,322], contributing to IFN-γ (NK cells and ILC1s), IL-17, and IL-22 (ILC3s) secretion at the local site of infection [321,323]. In vitro, NK cells restrict mycobacterial intracellular growth in mononuclear phagocytes in a contact-dependent manner [324,325]. TB patients present a weakened cytotoxic activity of NK cells [326,327,328,329,330], suggesting that active infection in humans might also impair NK cell function. This hypothesis is supported by the modified phenotypic and functional profiles of NK cells in TB endemic settings [331,332]. One possible molecular mechanism involves the PD-1/PD-1 ligands pathway, which is increased in NK cells from peripheral blood and pleural fluid of TB patients and which negatively correlates with IFN-γ production and degranulation [330].

Other approaches driven by pathogenic bacteria to suppress NK cell activity comprise the promotion of prostaglandin E2 (PGE2) production, which suppresses NK cell migration, cytokine production, and cytotoxicity [333,334] and the direct action of bacterial toxins. This is the case, for instance, for the *Bacillus anthracis* toxin [335] or the *Yersinia pestis* virulence protein YopM, which cause a global depletion of NK cells and affect the expression of IL-15 receptor [336]. An aspect that remains poorly understood but is becoming increasingly recognised is the role of ILCs in pathogenesis during bacterial infections, notably in the context of *Chlamydia muridarum*, *Salmonella enterica* Typhimurium, and *Helicobacter hepaticus* infections [337,338,339]. For instance, the accumulation of NK cells and ILC1s to a high density in the oviduct of *Chlamydia muridarum*-infected mice correlates with an enhanced pathology as measured by an increase of the oviduct weight [337].

Since their discovery, great advances have been made in the knowledge of the function and identity of ILCs [314,340], hence highlighting the need for further research on bacterial infections. Aforementioned evidence suggests that *Brucella* has developed yet unrecognised mechanisms of the immunosuppression of ILCs to favour its persistence. To unravel them, it is important to keep in mind that common experimental approaches using anti-NK1.1 and anti-asialo-GM1 antibodies to deplete NK cells also target ILC1s and ILC3s, making essential a better dissection of the role of each ILC subset in response to *Brucella* and other pathogens.

### 3.6. Host Lipids and Bacteria

For thousands of years, *M. tuberculosis* has co-evolved to adapt its life in the lipid-rich granuloma core, persisting in so-called foamy macrophages [341,342]. Mycobacteria accumulate host triacylglycerol in lipid droplets concomitant with the acquisition of a dormant-like phenotype inside hypoxic lipid-loaded macrophages [342]. This is just one example among many, making evident that the interaction and use of host lipids is essential for *M. tuberculosis* survival and persistence, which can be extended to numerous pathogens. In fact, there is a growing number of bacterial organisms known to use host lipids for internalisation into cells (such as *Chlamydia trachomatis* [343], *Francisella tularensis* [344], *Shigella flexneri* [345], *Coxiella burnetti* [346], and *Brucella* spp., as mentioned above), intracellular growth (as a source of energy but also to evade immune defences) and dissemination (including cholesterol-dependent cytolysins used by *Clostridium* species [347] and *Listeria monocytogenes* [348]).

Among the host lipids involved in immunity against pathogens, eicosanoids arise as attractive targets for the development of new therapies, since they compose a major bioactive lipid network with great implications in immune regulation and, thus, play critical roles during bacterial infections.

Host eicosanoids play an indisputable role in *Brucella* infections. Both *B. melitensis* and *B. abortus* LPSs induce COX-2 expression in the human monocyte cell line THP-1 in vitro, although at lower levels than those observed upon *E. coli* LPS stimulation [349]. A strong stimulation of the prostaglandins and leukotriene pathways is also elicited by *B. abortus* in murine bone-marrow derived DCs in vitro [350], and in vivo after intradermal, intranasal, and conjunctival inoculation [350]. Importantly, COX-2 inhibition with NS-398 reduces bacterial burden in draining lymph nodes at 8 days post-infection [350], suggesting that *Brucella* uses the prostaglandin pathway to survive and replicate during the acute phase of infection; whether it affects the chronicity of the infection remains unknown. Accordingly, the leukotriene B4, Lipoxin A4, and the prostaglandin I2 have been involved in brucellosis pathophysiology. 5-Lipoxygenase (LO)-deficient mice, which do not produce leukotriene B4 and lipoxin A4, present lower *B. abortus* loads in spleen and liver with milder liver pathology, as well as increased Th1 responses [351]. In macrophages, prostaglandin I2 inhibits *B. abortus* internalisation and attenuates pro- and anti-inflammatory cytokines production [352], while bacterial loads are decreased in prostaglandin I2-challenged and infected mice [352]. Altogether, *Brucella* exploits the eicosanoid pathway to escape the host immune response and survive.

Concerning TB, there has been growing interest in leukotrienes, prostaglandins, and lipoxins, as well as their interrelationship with each other, as critical determinants in the outcome of *M. tuberculosis* infection. Elevated levels of PGE2 are present in granulomas during the early phase of *M. tuberculosis* infection in mice [353]. Likewise, TB patients exhibit higher plasma concentrations of this eicosanoid as compared to healthy individuals [164,258]. Lower levels in TB patients correlate with severe clinical presentations [164]. In mice, an avirulent strain of *M. tuberculosis* induces PGE2 production; this PGE2 protects against cell necrosis by preventing damage to the inner mitochondrial membrane [354] and promoting rapid plasma membrane repair [355]. Another eicosanoid, the lipoxin A4 (LXA4), is the dominant lipid mediator produced by macrophages infected with virulent *M. tuberculosis*. LXA4 suppresses COX-2 expression and PGE2 synthesis, thus diverting the infected macrophage to a necrotic fate [354]. Manipulation of the infected macrophage cell death pathway utilised by *M. tuberculosis* through eicosanoid modulation constitutes a virulence strategy to survive in the host. In this scenario, macrophage apoptosis is proposed to be protective for the host given that it restricts bacterial growth [356,357], increases antigenic cross-presentation by DCs, and induces a specific Th1 adaptive response [358,359].

In addition, PGE2 at high concentrations is immunosuppressive for T cell-mediated immunity [164,353], affecting also monocyte and neutrophil functions [164], and contributes to the expansion of regulatory T cells [360] during infection with *M. tuberculosis*. This suggests that PGE2 might attenuate the excessive inflammatory immune response caused by the mycobacteria. In infected mice with an exacerbated type I response, treatment with zileuton, a 5-LO inhibitor, together with PGE2, leads to both decreased lung pathology and bacterial load [258], indicating that lipid mediators are interesting targets for therapeutic intervention in TB. Several on-going clinical trials aim at modulating the eicosanoid balance, either by inhibiting the COX-2 enzyme (as the case for non-steroidal anti-inflammatory drugs or other COX inhibitors) or by modulating E-type prostanoid (EP) receptors for prostaglandins.

These anti-TB host-directed therapies will be applicable to other chronic bacterial infections, in which different virulence strategies alter the expression of eicosanoid-specific biosynthetic enzymes [361]. Some yet unidentified effectors encoded by *Yersinia enterocolitica* participate in COX-2 signalling downregulation [362], whereas the exogenous addition of PGE2 results in a stronger inflammasome response, M1 macrophage polarisation, and decreased bacterial burden [362]. Similarly, the PGE2 produced in response to *Burkholderia pseudomallei* infection has harmful effects on the survival of infected mice [363], while intranasal infection with *Francisella tularensis* leads to the increased production of PGE2, which inhibits the generation of IFN-γ^+^ cells and promotes the generation of Th17 responses [364]. Altogether, it seems that eicosanoids shape the host immune response in a pathogen-dependent fashion and that intracellular bacteria subvert these pathways to survive. The timing, level, and balance between the different lipid mediators probably determine the final outcome; hence comprehensive and integrative studies are necessary to develop effective therapies for each pathogen.

This relevance of host lipids in host–pathogen interactions has led to a growing interest in the role of adipose tissue (AT) during infections, considering the specific features of these sites. The AT concerns 15–25% of the total body mass, distributed throughout the body. It serves as a reservoir for several bacteria, including *M. tuberculosis* [365], *Rickettsia prowazekii* [366] and potentially for *Brucella abortus* ([1] and Gonzalez-Espinoza et al., 2022, in preparation), allowing the pathogens to persist virtually anywhere in the body. Moreover, AT has been recognised as a persistence site for non-bacterial pathogens as well, such as human adenovirus Ad-36, influenza A virus, cytomegalovirus, HIV, and *Trypanosoma gondii* [367]. AT is a prototypic immunometabolic tissue in which immune and metabolic cells interact. Indeed, apart from adipocytes, it contains many different cell types, including macrophages, monocytes, lymphocytes, and DCs [368], that form tertiary lymphoid structures [369]. Thus, the immune response initiated at these sites during infectious challenges appears attractive for further investigations in persistent infections.

## 4. Perspectives

Chronic bacterial infections represent a huge burden in terms of public health (with elevated levels of morbidity and mortality) but have also important economic and social impacts. The lack of efficient vaccines and the weakness of current treatments that still rely on antibiotic therapy have evidently made the control of these diseases challenging, including brucellosis, whose eradication in many countries is still an aspiration.

**Figure 2:**
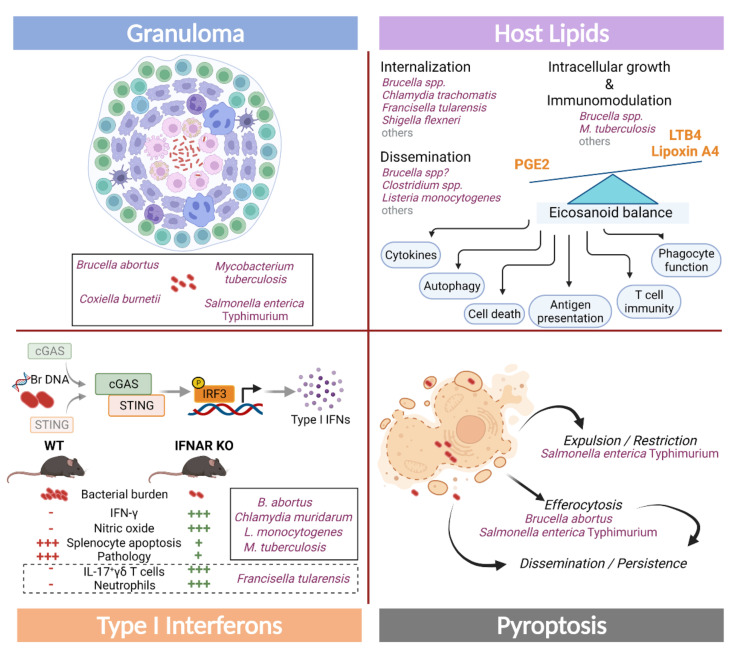
**Shared strategies by intracellular pathogenic bacteria during chronic infections.** Granulomas are focal aggregates of heterogeneous macrophages together with other immune cells at the infection site that form a physical barrier for escaping immune surveillance, but also offer a beneficial microenvironment for the bacteria (red rod), including *Brucella*, to remain viable for decades. The enriched anti-inflammatory milieu and the limitation of the positioning of effector immune cells provide a safe niche, and the constant movement of cells gives the opportunity for secondary infections. Interaction and use of host lipids by pathogenic bacteria play a central role for internalization, dissemination, intracellular growth and immunomodulation during chronic infection. The relative balance of eicosanoids modulates key processes, shown in blue boxes, and is essential for determining the pathogenesis and development of a proper anti-bacterial immunity. The role of type I interferons during bacterial infections is highly context-dependent with both beneficial and detrimental outcomes for the host. In infected macrophages, IRF3-mediated type I IFN production is promoted by c-GAS-STING recognition of *Brucella* DNA. IFNAR-deficient murine models of chronic infection have shown reduced bacterial burden and pathology, correlating with enhanced IFN-γ and nitric oxide production and diminished splenocyte apoptosis. Type I IFNs have been related to inhibit IL-17^+^γδ T cells and neutrophil expansion in *Francisella tularensis*-infected mice. The outcome of caspase-1/inflammasome-induced pyroptotic cell death is also highly dependent on timing and cell type. Pyroptotic intestinal epithelial cells are expelled into the lumen restricting Salmonella dissemination and replication. In contrast, efferocytosis of *Salmonella* or *Brucella* entrapped in pyroptotic macrophages shields the bacteria from the neutrophil respiratory burst, contributing to their persistence.

In this review, we have discussed various strategies shared by several intracellular bacteria to manipulate the host immune response and persist for long periods of time, as summarized in Figure 2. This might open the path to the development of targeted drugs, urgently needed given that long therapy with combinations of antibiotics may lead to antibiotic resistance but also to treatment failure or relapses. Moreover, antibiotic treatments may be a risk factor for all-cause and cardiovascular mortality in late adulthood [370]. In the past years, host-targeted therapies (HDT) have been proposed to be useful in combating bacterial infections and improving the efficacy of treatments. The strength of these strategies is that they act through host-mediated responses against the pathogen rather than acting directly against the pathogen, like traditional antibiotics. An example is to change the local environment in which the bacteria are found and making it less favourable for the pathogen to live and/or grow. Therefore, counteracting the stealthy behaviour of *Brucella* or its immunosuppressive properties represent good bases to develop innovative treatments to avoid chronic brucellosis.

One possibility may target the antigen presentation step, known to be inhibited by different means by *Brucella* as explained above. A proof of concept of this approach was brought by the restoration of *Brucella*-infected DC functionality after contact with activated Vγ9Vλ2 T cells, thus suggesting that this kind of cell-based treatment might be used to enhance immunity against pathogens [371]. The treatment of THP-1 monocytes with adrenal steroids, such as dehydroepiandrosterone (DHEA), also positively modulates costimulatory molecules, MHC-I and MHC-II, expression upon *B. abortus* infection, opening the potential for therapeutic interventions [372]. Overall, a better understanding of the key players during persistent infections, such as type I and III IFNs, or adipose tissues as reservoirs and sources of immune modulators, is an imperative for a proper delivery of targeted drugs to clear these intracellular bacterial pathogens.

Another HDT approach suggested in the context of TB infection is the disruption of the granuloma, as an opportunity to improve the ability of crucial immune cell types to reach the bacterial niche, recognise, and eliminate bacteria [373,374]. This type of scheme might also enhance the penetration of drugs at the right place, since many of them have demonstrated effectiveness in vitro but failed to translate into new clinical antibiotics [373]. However, dissemination of the disease and death may also result from granuloma disruption. Anti-TNF drugs have been indeed associated with risk for reactivation of latent tuberculosis infection and disease progression [375,376,377]. Thus, a better characterisation of all the components of *Brucella* granuloma as well as their spatial organization in the acute and chronic phase of the disease is absolutely necessary and will help to choose pertinent therapeutic targets.

As summarized in this review, the evasion and immunosuppressive mechanisms evolved by *Brucella* comprise multiple mechanisms and players. In brucellosis, microRNAs display a differential expression signature upon infection [378]. This suggests that microRNAs might contribute to the transition towards chronicity by regulating specific host processes during brucellosis (reviewed elsewhere [379]). In the design of an efficient vaccine, the generation of tissue-resident memory T cells is highly desirable as an ideal first line of defence and in light of their remarkable role against pathogens in the long-term [380]. Additionally, the promotion of T cell exhaustion upon *Brucella* infection [166,381] requires more consideration.

The advent of next-generation sequencing technologies and single-cell approaches will undoubtfully help to identify specific subpopulations with key immunoregulatory properties, molecular signatures, or possible host and bacterial targets. Single-cell RNA sequencing (scRNA-Seq) has become a powerful tool to understand the complex profiles of immune cells during infections allowing to detect not only gene expression differences, but also to identify distinct subpopulations among cell types and to map cellular interacting networks. Thus, within the limitations of a BSL3 facility, multi-organ analysis might reveal novel interactions and allow the detailed analysis of understudied subsets, such as ILCs during chronic brucellosis. scRNA-Seq may also be applied to decipher the immune repertoire of B and T cells in light of the mentioned mechanisms to impede the initiation of adaptive responses, helping to accelerate the development of specific vaccines. Characterisation of the impact in a natural or experimental host of *Brucella* mutants obtained by CRISPR/Cas9 technology will be highly facilitated too. The further combination of scRNA-Seq with spatial transcriptomics has been employed to analyse immune landscapes associated with histopathological features in chronic infections, such as TB and trypanosomiasis [382,383]. This methodology might also help to uncover the tissue architecture and cell interactions of the *Brucella*-induced granuloma, as well as tertiary lymphoid structures observed in AT. The integration of heterogeneous data from multi-omics studies will require powerful bioinformatic tools as well; the development of artificial intelligence approaches might pave the way to the design of novel drugs and better diagnosis [384,385].

Finally, the mouse model of infection has been one of the most valuable tools for unravelling the subversion mechanisms of the immune response by *Brucella*, thanks to its cost-effectiveness and simplicity of genetic manipulation. Nevertheless, differences between this model and the animal host or the humans in response to *Brucella* have already been pointed out [95], emphasizing the need to take any direct extrapolation with caution. The effectiveness of HDT is likely to depend on disease phenotype and on the timing of its use [386]. Thus, further studies should aim not only at taking into account host species but also at identifying endotypes, defined as distinct patient populations with specific molecular profiles and given metabolic, epigenetic, transcriptional, and immune phenotypes. Improving our understanding of the disease characteristics in patients with acute and chronic brucellosis with diverse outcomes is also mandatory. As such, current technologies might guide the identification of new biomarkers of these endotypes and allow a better and personalised treatment design.

This review has raised several questions that remain open and should be answered in the coming years, among them the following: How can we circumvent the *Brucella* intracellular niche for its elimination? Which *Brucella* effectors modify the host metabolism in diverse cell types? What are the roles of type I and III IFNs at the different stages of the infection? How can adipose tissue be bacterial reservoirs and immunosuppressive hubs? Moreover, how can we target them?

An in-depth comprehension of the immune mechanisms that pathogenic bacteria exploit to persist in chronic infections will enable a better control of these diseases, including brucellosis, and even improve our understanding of the function of the immune system per se.

## Data Availability

Not applicable.
